# On the Chemical Potential of Many-Body Perturbation Theory in Extended Systems

**DOI:** 10.1021/acs.jctc.2c01043

**Published:** 2023-02-15

**Authors:** Felix Hummel

**Affiliations:** Institute for Theoretical Physics, TU Wien, Wiedner Hauptstraße 8-10/136, 1040 Vienna, Austria

## Abstract

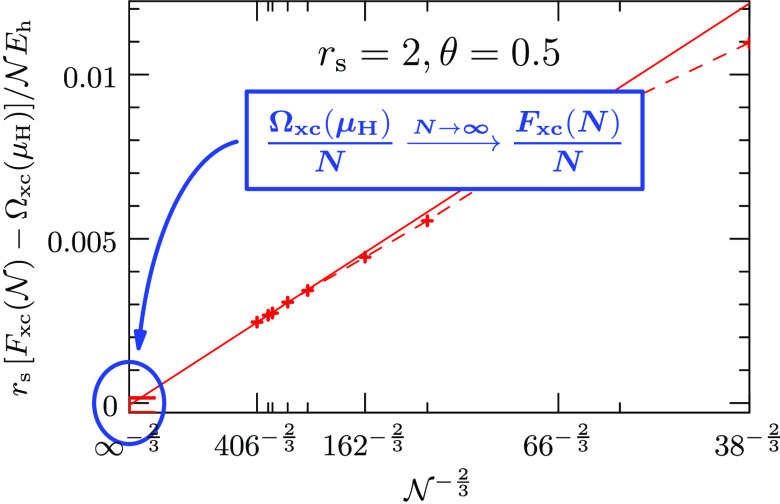

Finite-temperature many-body perturbation theory in the grand-canonical ensemble is fundamental to numerous methods for computing electronic properties at nonzero temperature, such as finite-temperature coupled-cluster. In most applications it is the average number of electrons that is known rather than the chemical potential. Expensive correlation calculations must be repeated iteratively in search for the interacting chemical potential that yields the desired average number of electrons. In extended systems with mobile charges the situation is particular, however. Long-ranged electrostatic forces drive the charges such that the average ratio of negative and positive charges is one for any finite chemical potential. All properties per electron are expected to be virtually independent of the chemical potential, as they are in an electric wire at different voltage potentials. This work shows that per electron, the exchange-correlation free energy and the exchange-correlation grand potential indeed agree in the infinite-size limit. Thus, only one expensive correlation calculation suffices for each system size, sparing the search for the interacting chemical potential. This work also demonstrates the importance of regularizing the Coulomb interaction such that each electron on average interacts only with as many electrons as there are electrons in the simulation, avoiding interactions with periodic images. Numerical calculations of the warm uniform electron gas have been conducted with the Spencer–Alavi regularization employing the finite-temperature Hartree approximation for the self-consistent field and linearized finite-temperature direct-ring coupled-cluster doubles for treating correlation.

## 1 Background

In the warm-dense matter (WDM) regime a large number of many-body states are thermalized. Also, the density is sufficiently large to require a quantum mechanical treatment of the electrons interacting with each other. WDM conditions are found, for instance, during inertial confinement fusion (ICF), in the metallic phase of hydrogen in gas giants, or in matter interacting with high intensity laser fields.^1^ Even at room temperature the thermal energy must be considered to be large compared to the vanishing band gap of bulk metals.

The mobility of electrons at warm-dense conditions poses challenges for *ab initio* simulations of extended systems that are absent in zero-temperature calculations. Unlike at zero temperature, the number of electrons in a volume of fixed shape fluctuates. Periodic boundary conditions, usually employed in extended systems, cannot model such fluctuations at length scales beyond the size of the simulation cell. In reality, the charges would move from one cell to the neighboring cell, keeping the average charge constant. Under periodic boundary conditions, on the other hand, charges can only appear or disappear simultaneously in all periodic images of the simulation cell. However, configurations of periodically repeating net-charged cells are not allowed due to the diverging electrostatic energy per volume. There are mainly two methods in current state-of-the-art *ab initio* simulations at warm-dense conditions to circumvent this difficulty: (i) The simulation is done in the canonical ensemble where electrons are not permitted to enter or leave the simulated volume. While this ensures charge neutrality it also reduces the number of possible configurations, affecting the system’s entropy.^2^ Path-integral quantum Monte Carlo (PIQMC) calculations are usually conducted in the canonical ensemble.^3,4^ (ii) Another possibility is to disregard the parts of the electrostatic interaction stemming from the average electron and background densities, thus removing the divergence. This allows for grand-canonical simulations with a fluctuating number of electrons including its effect on the entropy. Perturbation-theory-related calculations usually apply this method^5,6^ following the work of Kohn and Luttinger, in particular the assumption for arriving at eq 20 in ref (7). A physical justification for this procedure would be if the fluctuations of the positive background were fully correlated with the fluctuations of the electrons. Different mobilities of electrons and ions, however, question this assumption.

In this work, a third alternative is studied to treat long-range electrostatic interactions under periodic boundary conditions. Liang and co-workers^8^ have studied classical simulations of mobile electrostatically interacting particles under periodic boundary conditions. They look at the pair correlation function and observe the theoretically expected Debey–Hueckel screening at long distances only under two conditions: (i) when simulating in the grand-canonical ensemble, and (ii) when limiting the range of the electrostatic interaction, such that the particles do not interact with all of their own periodic images. Here, the Coulomb interaction is truncated spherically such that the sphere’s volume agrees with the volume of the simulation cell. This modification is called *regularization* of the Coulomb interaction and it turns divergent terms into finite terms. Care must be taken that the infinite-size limits of the computed quantities are not dependent on the details of the regularization scheme, as discussed in section 2.

A spherical truncation scheme has already been developed by Spencer and Alavi^9^ to avoid spurious Fock-exchange interactions of the electrons with their periodic images for zero-temperature calculations as an alternative to other methods treating the occurring integrable singularity.^10,11^ This work applies the truncation scheme to all parts of the electrostatic interaction in the self-consistent field calculations, as well as in the subsequent correlation calculation. Other regularization schemes that limit the interaction range are also possible, such as the minimal image convention (MIC) for atom centered orbitals, or the Wigner–Seitz truncation scheme.^12,13^ For point-like charges the spherical truncation is not continuous which may pose difficulties when considering different atomic configurations.

### Related Work

Finite-temperature many-body perturbation theory (FT-MBPT) offers an elementary framework for *ab initio* calculations of WDM.^5,6,14−16^ Numerous approximation schemes employ thermal MBPT, such as thermal second-order MBPT,^17−20^ finite-temperature random phase approximation,^21−24^ Green’s function based methods,^25,26^ as well as some finite-temperature generalizations of coupled-cluster methods.^27−30^ An alternative formulation of the coupled-cluster methods has been brought forward recently in the framework of thermo-field dynamics.^31−33^ Finite-temperature perturbation theory is originally formulated in the grand-canonical ensemble; however, formulations in the canonical ensemble exist.^34,35^ Equally, thermo-field dynamics can be employed in the canonical ensemble.^36^

Analogous to *ab initio* calculations at zero-temperature, thermal Hartree–Fock and density functional theory (DFT) calculations are among the most widely used methods serving also as self-consistent field (SCF) reference for FT-MBPT.^37−39^ For DFT it is in general not sufficient to use a zero-temperature exchange-correlation functional and introduce temperature merely by smearing. Temperature must be a parameter of the exchange-correlation functional.^40^ At higher temperatures, a large number of one-body states is occupied with non-negligible probabilities. Orbital-free density functional theories (ofDFT) aim at mitigating this with functionals that do not depend on the usual Kohn–Sham orbital description of DFT.^41,42^ Canonical or grand-canonical full configuration interaction methods can be used for benchmarking more approximate theories.^43^ Finally, various forms of Monte Carlo methods approach the finite-temperature many-body problem in complementary ways as they exhibit entirely different error sources. They include path-integral Quantum Monte Carlo (PIQMC) calculations,^3,4^ Density Matrix Quamtum Monte Carlo (DMQMC) calculations,^44−46^ and Auxiliary Field Quantum Monte Carlo (AFQMC) calculations.^47−49^ While PIQMC calculations are usually conducted in the canonical ensemble, DMQMC and AFQMC calculations have also been done in the grand-canonical ensemble. High accuracy calculations of the warm uniform electron gas are of particular interest since they can serve for accurate temperature-dependent parametrizations of DFT exchange-correlation potentials.^50−52^

The Kohn–Luttinger conundrum is also closely related to this work. It states that the infinite-size zero-temperature limit of finite-temperature many-body perturbation theory not necessarily agrees with the infinite-size limit of zero-temperature many-body perturbation theory. In the common approach where the zero-momentum part of the electrostatic interaction is disregarded, certain terms called *anomalous diagrams* affect both, the chemical potential and the grand potential. It has been shown that their contributions cancel in the zero-temperature limit of the free energy under isotropic conditions^7^ and later more generally.^53,54^ Hirata and co-workers developed a perturbation theory that simultaneously produces order-by-order corrections to the grand potential and the chemical potential, while fixing the expected number of electrons.^55,56^ Employing the number-conserving perturbation theory and treating possible degeneracies of the SCF reference in the low-temperature limit,^57^ they overcome the conundrum.^58^ With the method of this work the situation is different and will be discussed at the end of subsection 2.4. Further discussions can be found in refs (18−20 and 59).

## 2 Methods

Let us now develop the regularization approach for the prototypical warm-dense system: the warm uniform electron gas (UEG). The UEG is a model of a metal, where the positive ions of the lattice are replaced by a static homogeneous positive background charge. It has a vanishing band gap in the infinite-size limit and thus qualifies for a warm-dense system at all nonzero temperatures. All properties of the warm UEG depend only on the thermodynamic state, specified by its density and temperature. The density is usually given in terms of the Wigner–Seitz radius *r*_s_ in atomic units, such that the volume of a sphere with radius *r*_s_ corresponds to the average volume per electron. It is also convenient to specify the temperature in terms of the dimensionless ratio θ = *k*_B_*T*/ε_F_, where *k*_B_*T* is the average thermal energy and ε_F_ = *k*_F_^2^/2 is the Fermi energy of a free spin-unpolarized (paramagnetic), infinite electron gas at the corresponding density and at zero temperature with *k*_F_^3^ = 9π/4*r*_s_^3^. This defines a natural temperature scale where different densities can be compared to each other more directly.

**Figure 1:**
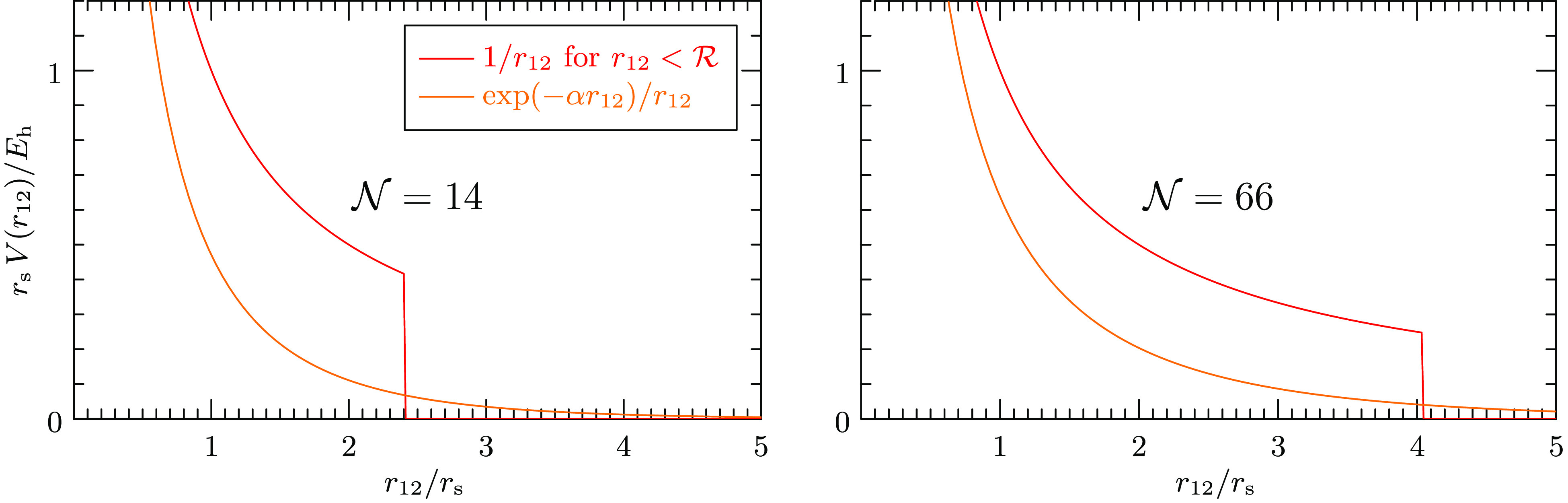
Illustration of the Spencer–Alavi (red) and the Yukawa (orange) regularization of the electrostatic interaction for different system sizes . The respective regularization parameter  or α is coupled to the system size  such that the scope of the interaction agrees with the system volume .

The UEG is modeled by a finite cubic box of length  under periodic boundary conditions having the volume . It contains a homogeneous positive charge density with a total charge of  elementary charges, which is considered fixed. In the grand-canonical ensemble the number of electrons in the system is not fixed but rather fluctuates around its expectation value which depends on the chemical potential μ. Later, μ will be chosen such that the expected number of electrons  equals, or is close to, the number of positive charges . To treat the diverging electrostatic interaction, the Spencer–Alavi or the Yukawa regularization of the electrostatic interaction is used. The Spencer–Alavi interaction is given by the usual Coulomb interaction 1/*r*_12_ for the distance between two electronic coordinates  that lie within a sphere of radius . The interaction is zero otherwise. The truncation radius  is coupled to the system size  and the simultaneous limit  is studied. The kernel of this interaction within a sum over momenta is . For finite  the kernel is also finite at *q* = 0 and evaluates to . With this choice the interaction “sees” on average  electrons. In the limit  it reduces to the usual electrostatic interaction 1/*r*_12_ for all distances.^9^ The Yukawa regularization is also studied for comparison. It is given by exp(−*αr*_12_)/*r*_12_ in real space and by  in momentum space in a sum over states. The regularization parameter α is chosen such that the weighting function exp(−*αr*_12_) integrates to , which means that each electron “sees”  electrons. With this choice, the regularization parameter is coupled to the system size satisfying  and the limit  is studied. For numerical computations the Yukawa regularization is an inefficient choice since asymptotic behavior usually only sets in at impracticably large system sizes. While it is not useful for retrieving absolute free energies, the difference between free energies reaches asymptotic behavior for the system sizes studied in this work. Figure 1 illustrates the regularized electrostatic interaction energy between unit charges at a distance *r*_12_ for the employed regularization schemes for different system sizes.

All states are expanded in antisymmetrized products of one-electron wave functions that are eigenfunctions of the single-electron kinetic operator –**∇**^2^/2 under periodic boundary conditions. The normalized eigenfunctions are the plane waves commensurate with the box length
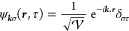
1with  and where σ, τ ∈ {*↑*, *↓*} denote the spin coordinate of the wave function and the electron, respectively. With the operator  creating an electron in the state ***k***, σ and  annihilating it, the electronic Hamiltonian of the modeled UEG reads
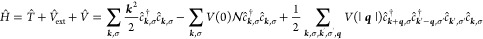
2It consists of three terms: the kinetic term *T̂*, the electron–background interaction  and the electron–electron interaction , respectively. Exact diagonalization of the Hamiltonian is infeasible except for very limited system sizes. This work shall employ the approximation approach of computational materials science, where one first performs a self-consistent field calculation, followed by an approximation of the correlation based on the SCF result. In accordance with the common workflow of random phase approximation (RPA) calculations for low-band gap systems, the SCF only employs the Hartree approximation rather than Hartree–Fock and exchange is considered at first order non-self-consistently.^60^ The finite temperature correlation contributions are estimated by a linearized form of the direct-ring coupled-cluster doubles approximation.

### 2.1 Self-Consistent Field in the Hartree Approximation

In the self-consistent field approach the two-body operator in the electron–electron interaction  is partially contracted to a one-body interaction.^37^ In the Hartree approximation only the direct contraction is considered and the resulting one-body operator is given by
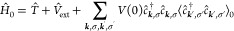
3where  denotes the one-body thermal equilibrium expectation value of the operator , defined by
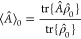
4with the (non-normalized) one-body density matrix
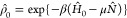
5All terms in eq 3 are diagonal in the chosen basis so we can immediately write the equations for the eigenvalue of each state *i* = (***k***_*i*_, σ_*i*_)
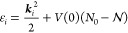
6with , Introducing the notation ε_*i*_ = ***k***_*i*_^2^/2 + Δε, we have to find a shift of eigenenergies Δε, uniform for all states, satisfying the nonlinear equation
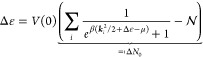
7for the given thermodynamic state point . So far, the number of positive charges  is an independent parameter. The quantity Δ*N*_0_ denotes the net-negative charge of the system in the noninteracting approximation. Note that it may differ from zero for charge neutral systems as the fully interacting  may differ from its noninteracting approximation . Having solved eq 7 for Δε we can evaluate the noninteracting grand potential
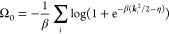
8with the *effective chemical potential η* ≔ μ – Δε. If we want to compare energies per electron for different system sizes we also need to account for the background–background interaction energy, which is independent of the electronic degrees of freedom. Furthermore, pairwise interactions in Ω_0_ are double-counted in the SCF. Accounting for both contributions yields the mean-field grand potential in the Hartree approximation
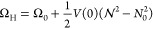
9Note that the expected number of electrons in the Hartree approximation *N*_H_ = −∂_μ_Ω_H_ equals the expected number of electrons of the noninteracting system *N*_0_ = −∂_μ_Ω_0_, which is given by the sum *∑*_*i*_*n*_*i*_ of noninteracting occupancies 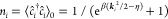


The Hartree grand potential Ω_H_(μ) is a function of the chemical potential μ. Of particular interest is the chemical potential μ_H_ for which the expected number of electrons in the Hartree approximation *N*_H_ matches the number of positive charges . This chemical potential is chosen for determining the Hartree free energy from a Legendre transformation . For this particular chemical potential, the solution of the Hartree equation Δε = 0 follows trivially from eq 7 and the effective chemical potential η_H_ = μ_H_ – Δε equals the Hartree chemical potential.

#### A Rough Estimate of the Hartree Self-Consistent Field

Before turning to the other contributions to the grand potential, it is interesting to estimate the Hartree solution Δε for chemical potentials μ close to the chemical potential μ_H_, which satisfies . To this end, we expand the expected number of electrons *N*_H_ = −∂_μ_Ω_0_ as a function of the effective chemical potential η = μ – Δε at the Hartree effective chemical potential η_H_ = μ_H_, where Δε = 0. The expansion reads
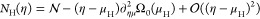
10where  with the shorthand notation . We can approximate the difference  between the expected number of electrons in the SCF calculation and the number of positive charges to first order in (η – μ_H_) by
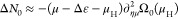
11We further assume that in the warm-dense-matter regime the term *∑*_*i*_*n*_*i*_(1 – *n*_*i*_) scales linearly with system size. Together with eq 7 Δε = *V*(0)Δ*N*_0_ we are now in the position to approximately solve eq 11 for Δ*N*_0_:
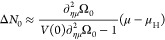
12Inserting Δ*N*_0_ into eq 7 and expanding in powers of  for large  finally yields
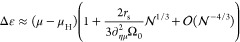
13
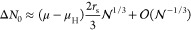
14where we inserted  for the Spencer–Alavi truncated Coulomb kernel. Interestingly, the  terms in the above equations are exactly linear in (μ – μ_H_), although Δ*N*_0_ may have terms depending on higher orders of (μ – μ_H_). This stems from the relation , which is exactly linear. In case of the Yukawa regularization, *V*(0) is  which gives a prefactor of 6^2/3^*r*_s_/3 instead of 2*r*_s_/3 in both of the above equations.

This is an essential result. For a finite deviation of the chemical potential μ from the noninteracting chemical potential μ_H_ = μ_0_, the expected number of excess electrons  of the entire system diverges as . It grows proportionally to the range  of the regularized Coulomb interaction. However, the expected number of excess electrons per positive charge always converges to one as  and the electrostatic energy of the system per electron Δε asymptotically reflects the change of the chemical potential. When changing the chemical potential, the relaxed self-consistent field eigenenergies asymptotically change in the exact same way for large enough system sizes. The numerical results in subsection 3.1 indicate that this behavior already sets in for relatively low system sizes. Since only the difference of the chemical potential μ and the eigenenergies ε_*i*_ = ***k***_*i*_^2^/2 + Δε enter in subsequent calculations, size-intensive observables will be asymptotically independent of the choice of μ in the thermodynamic limit. At warm-dense conditions, any finite choice is acceptable, including μ = 0, which is the classical definition of the chemical potential of electrons in a grounded conductor that can supply or absorb any number of electrons.

#### Decoupling the Infinite-Range Limit of the Coulomb Interaction

Instead of the simultaneous limit of , studied in this work, one could also perform the infinite-range limit  before performing the infinite-size limit . An estimate, analogous to eqs 13 and 14, yields 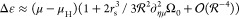 and 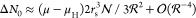
respectively. With increasing range , deviations become increasingly expensive and one arrives asymptotically at the canonical ensemble for exactly  electrons, where the chemical potential μ plays no role anymore. However, classical systems of charged particles exhibit larger finite-size errors when simulating in the canonical ensemble compared to simulating in the grand-canonical ensemble with the range of the Coulomb interactions coupled to the size of the simulation cell.^8^ Thus, this work pursues the latter approach.

#### Using Fixed Instead of Relaxed Orbitals

If one chooses to work with a fixed set of orbitals and eigenenergies for various values of the chemical potential μ, the electron–background interaction 
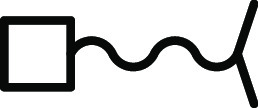
 and the electron–electron density interaction 
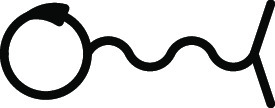
 do not cancel for μ deviating from μ_H_. There are terms of order *n* in the perturbation series whose contributions scale as . They are not extensive for each *n* and alternating in sign. This can be cured by summing the interactions to infinite order, which is equivalent to performing an SCF calculation at the modified chemical potential.^61^

#### Fluctuations of the Number of Electrons

From  we can also estimate the variance *δN*_H_^2^ of the fluctuations of the number of electrons in the SCF calculation for large  by
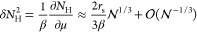
15This agrees qualitatively with classical charge fluctuations *δN*_C_^2^ on the surface of a grounded conducting sphere of radius , found from the equipartition theorem
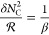
16Note that we need to consider the response of the one-body energies to changes of the chemical potential for computing derivatives of the grand potential beyond first order, such as *∂N*_H_/*∂μ* = −∂^2^Ω_0_/*∂μ*^2^ in eq 15. For comparison, a fixed density matrix  that separates into a product of one-body density matrices is only capable of describing electron-number fluctuations of the form , which is proportional to  rather than to  at warm-dense conditions.

### 2.2 First-Order Exchange

The self-consistent field approximation is crude but computationally efficient. To improve on the approximation, finite temperature many-body perturbation theory (FT-MBPT) offers an expansion of the grand potential in powers of the difference  between the true Hamiltonian *Ĥ* and the self-consistent field Hamiltonian . Having employed the Hartree approximation for the SCF, the leading order term is the first-order exchange term
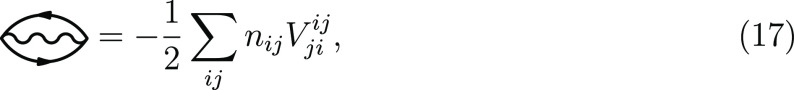
17where we again use compound indices *i* = (***k***_*i*_, σ_*i*_) and *j* = (***k***_*j*_, σ_*j*_) to denote the spatial and spin components of the respective spin-orbitals. We also employ the shorthand notation *n*_*ij*..._ = *n*_*i*_*n*_*j*_... for products of one-body occupancies. *V*_*sr*_^*pq*^ denotes the components of the electron–electron interaction operator in the basis of the plane-wave spin-orbitals such that 
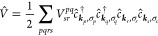
For a translationally invariant, isotropic interaction the components read
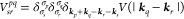
18The first-order exchange term with non-Hartree–Fock orbitals is often referred to as *exact exchange* (EE). It is given by
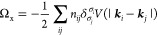
19Adding the first-order exchange contribution Ω_x_ to Ω_H_ yields the improved Hartree-exchange approximation Ω_Hx_.

### 2.3 Linearized Direct-Ring Coupled Cluster

Let us now turn to correlation and exchange effects beyond first order. Here, it is treated at the level of linearized direct-ring coupled-cluster doubles (ldrCCD) theory.^30^ A truncation of the perturbation expansion at any finite order *n* diverges as  for the uniform electron gas for low temperatures *T* in the infinite-size limit. On the other hand, summing over the so-called ring terms up to infinite order *n* yields convergent results at zero temperature in the limit  after *n* → *∞*.^62,63^ The analogous ring-term summation of finite-temperature MBPT also yields convergent results for all temperatures although its limit *T* → 0 might differ from the result obtained from a zero-temperature theory. We desire a theory with such a convergence behavior for *T* → 0, at least in principle. ldrCCD is one of the simplest theories providing this resummation of the ring terms. It contains all ring terms that can be formed with exactly two particle/hole pairs and additionally contains their corresponding screened-exchange terms. It is determined by the finite-temperature linearized direct-ring coupled-cluster amplitude integral equations
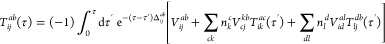
20with Δ_*ij*_^*ab*^ = ε_*a*_ – ε_*i*_ + ε_*b*_ – ε_*j*_ and where we now also need products of vacancy and occupancy probabilities, denoted by . eq 20 can also be given in terms of diagrams
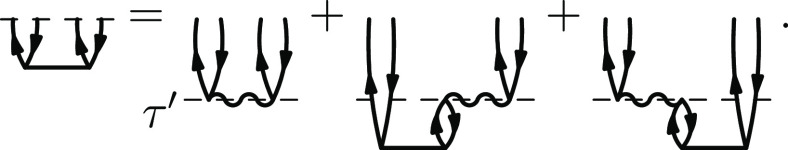
With the solutions of the amplitude functions *T*_*ij*_^*ab*^(τ), satisfying eq 20 on the interval τ ∈ [0, β] with the initial conditions *T*_*ij*_^*ab*^(0) = 0, the ldrCCD grand potential can be evaluated from
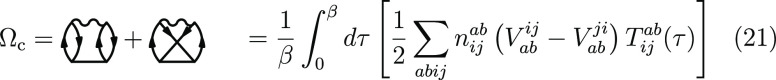
21with . All indices iterate in principle over the infinite number of plane wave states. Practical truncation schemes are discussed in section 3.3. The linear system of coupled integral equations in eq 20 can be solved by diagonalizing an effective particle/hole interaction *H̃*, analogous to the Tamm–Dancoff approximation of the Casida equations at zero temperature. The effective particle/hole interaction reads
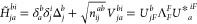
22which, interpreting the indices (*b*, *j*) as a compound row index and the indices (*a*, *i*) as a compound column index, is a hermitian matrix and thus permits a real-valued eigendecomposition. We can then transform the electron repulsion integrals with and without exchange into the space of eigenmodes
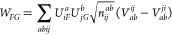
23
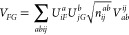
24and finally retrieve the ldrCCD approximation of the correlation grand potential from
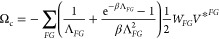
25with Λ_*FG*_ = Λ_*F*_^*F*^ + Λ_*G*_^*G*^ and  denoting the conjugate transpose.^30^

### 2.4 Free Energies

So far, we have discussed all considered contributions to the grand potential
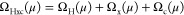
26as a function of the thermodynamic state point in the grand-canonical ensemble, in particular of the chemical potential μ. The number of positive charges  is merely a system parameter. We are, however, interested in the free energy  of the charge-neutral system where the expected number of electrons *N*_Hxc_≔ −∂_μ_Ω_Hxc_ equals the fixed number  of positive charges. It is found from the Legendre transformation
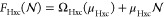
27where μ_Hxc_ satisfies the charge-neutrality condition for the Hartree-exchange-correlation grand potential . The final quantity of interest is the exchange-correlation (xc) free energy *F*_xc_ = *F*_Hxc_ – *F*_H_ beyond the free energy of the self-consistent field solution , where μ_H_ satisfies the charge-neutrality condition for the Hartree grand potential . Note that in general the Hartree-exchange-correlation chemical potential μ_Hxc_ differs from the Hartree chemical potential μ_H_, which is the noninteracting chemical potential.

#### A Rough Estimate of the Exchange-Correlation Free Energy

Let us now estimate the behavior of μ_Hxc_ and *F*_xc_ for large system sizes . We start by looking at the charge-neutrality condition , for the Hartree-exchange-correlation chemical potential μ_Hxc_. From eq 26 we can immediately write the expected number of electrons as −∂_μ_Ω_H_ – ∂_μ_Ω_xc_, where −∂_μ_Ω_H_ = *N*_H_ is the expected number of electrons in the Hartree approximation at the interacting chemical potential μ_Hxc_, which differs from  for μ ≠ μ_H_. At the end of subsection 2.1 we have estimated that it behaves as  for sufficiently large , according to eq 14. From eqs 19 and 25 it follows that the only terms that depend on μ in the remaining contribution Ω_xc_ are the occupancy and vacancy expectation values  and *n*^*a*^ = 1 – *n*_*a*_, respectively. The expectation values *n*_*i*_ depend only on the difference ε_*i*_ – μ between the eigenenergies ε_*i*_ = ***k***_*i*_^2^/2 + Δε and the chemical potential μ, where Δε is the shift of eigenenergies, uniform for all states *i*, found from the self-consistent field solution for the interacting chemical potential μ_Hxc_. Using the notion of the effective chemical potential η = μ – Δε introduced in subsection 2.1, we can write the derivative with respect to the chemical potential in terms of a derivative with respect to the effective chemical potential from the chain rule
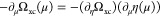
28We have already estimated the asymptotic behavior of Δε in eq 13 from which we can find the behavior of ∂_μ_η(μ) for large :
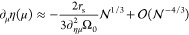
29Note that −∂_*ημ*_^2^Ω_0_ = β∑_*i*_*n*_*i*_^*i*^ scales linearly with  under warm-dense conditions. Similarly, since ∂_η_*n*^*a*^ = −*βn*_*a*_^*a*^, we can also assume that −∂_η_Ω_xc_ scales at most linearly with the system size  under these conditions. Collecting all contributions to the expected number of electrons gives
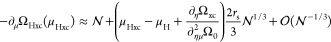
30where the fraction inside the parentheses does not depend on  asymptotically. Remarkably, this means that the expected number of electrons per positive charge  converges asymptotically to one for large system sizes for any choice of the chemical potential μ_Hxc_. Still, the absolute deviation of *N*_Hxc_ from  does depend on μ_Hxc_ and scales as  with the number of positive charges . From this deviation we can approximately solve the charge-neutrality condition to find the Hartree-exchange-correlation chemical potential:
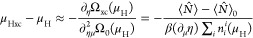
31Although the expected number of electrons per positive charge converges to one for any chemical potential in the thermodynamic limit, there is a nonvanishing deviation from the noninteracting chemical potential μ_H_ required if also the absolute expected number of electrons *N*_Hxc_ should match the number of positive charges for large .

Knowing the asymptotic behavior of the interacting chemical potential μ_Hxc_ we can now estimate the free energy for large system sizes. For that purpose, we expand the Hartree-exchange-correlation free energy at the noninteracting chemical potential in eq 27 in powers of the difference (μ_Hxc_ – μ_H_), which we have found to be finite but approximately independent of :
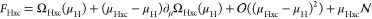
32Subtracting the Hartree free energy  we arrive at an estimate of the exchange-correlation free energy expansion
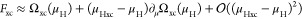
33Our estimate of ∂_μ_η in eq 29 is approximately independent of μ. Therefore, the higher derivatives of the exchange-correlation grand potential occurring in the above expansion are estimated to be of the form  and they thus scale at most as .

Using eq 28 for ∂_μ_Ω_xc_ and inserting the estimate for (μ_Hxc_ – μ_H_) from eq 31 finally gives us an estimate of the exchange-correlation chemical potential for large 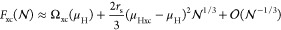
34In the thermodynamic limit the exchange-correlation grand potential per electron, evaluated at the noninteracting chemical potential, is estimated to agree with the exchange-correlation free energy per electron , found at the interacting chemical potential. As for the SCF estimates, using the Yukawa regularization yields a prefactor of 6^2/3^*r*_s_/3 instead of 2*r*_s_/3 in the above equation.

This is the main result of this work, and the numerical studies in the following section show that this asymptotic estimate applies already at relatively small system sizes in the uniform electron gas for the densities and temperatures considered. For finite system sizes , eq 34 relates the difference between Ω_xc_(μ_H_) and  to the difference between the interacting and the noninteracting chemical potential. The latter converges faster with system size and this relation permits an estimate of the remaining finite-size error in Ω_c_ for the thermodynamic limit extrapolation. One can also employ eq 31 to estimate the interacting chemical potential μ_Hxc_ from a correlation calculation at a noninteracting chemical potential if the derivative with respect to η can be found efficiently.

#### Comparison with the Madelung Technique and the Kohn–Luttinger Conundrum

This work examines the simultaneous limit of the interaction range  and the system size . The scope of the electrostatic interaction is limited to the size of the system . This is the key ingredient for the free energy per electron to be asymptotically independent of the chemical potential. A spherically truncated interaction and the Yukawa regularized interaction are studied in detail in this work. Other truncation schemes, such as the minimal image convention or the Wigner–Seitz truncation, are also expected to yield this behavior, albeit with different prefactors. If the infinite-range limit  is taken before the thermodynamic limit , the ensemble reduces to the canonical ensemble, as discussed at the end of subsection 2.1, which is expected to exhibit larger finite-size errors already in classical systems.^8^

A common alternative to limiting the scope of the electrostatic interaction is the Madelung technique. It considers the interaction energy ξ < 0 of an elementary point charge with its own periodic images and a neutralizing background charge for the zero-momentum part of the Coulomb interaction *V*(*q* = 0) = −ξ. For other momenta ***q*** with *q* = |***q***| > 0, the standard Coulomb interaction  is employed.^64^ This technique was developed for Monte Carlo simulations in the canonical ensemble and assumes a charge-neutral simulation cell under periodic boundary conditions. However, it has also been applied to finite temperature MBPT calculations in the grand-canonical ensemble,^28,49^ where charge-neutrality occurs only on average unless the fluctuations of electrons and positive charges are fully correlated—an unlikely scenario considering their different masses. Otherwise, this choice for the Hamiltonian is an approximation. In the limit of infinitely large simulation cells, the Madelung term vanishes ξ → 0 and one arrives at the assumption *V*(*q* = 0) = 0 that Kohn and Luttinger make to arrive at eq 20 in ref (7).

In this work they find that the infinite-size and zero-temperature limit of the grand potential in metals depends on the order of the limits and reduces to the value produced by zero-temperature MBPT only for  after *T* → 0 and if the zero-temperature limit of the SCF reference is nondegenerate. In the paramagnetic case, all orbitals of a common eigenenergy must be asymptotically doubly occupied or not at all. For the simple-cubic UEG this only applies to system sizes of  This discontinuity is called Kohn–Luttinger conundrum, although the authors never used this term. In the reverse order, the result differs by a non-zero value, stemming from so-called anomalous terms.^7^ Such terms contain orbitals that are simultaneously particles and holes and these terms are nonvanishing if the density of states at the Fermi surface is nonvanishing. For an electron–electron interaction satisfying the assumption *V*(*q* = 0) = 0 they also show that the correlation contribution of the anomalous terms to the chemical potential exactly counteracts the anomalous terms in the grand potential when regarding the Legendre transformed free energy for isotropic systems.

With the method proposed in this work, the situation is different. For a fixed finite *T* > 0, the free energy per electron is asymptotically independent of the chemical potential for  according to eq 34. Correlation corrections to the chemical potential play no role for the free energy per electron and contributions from anomalous terms to the exchange-correlation grand potential are not canceled. Taking the limit *T* → 0 after  may yield a different result than  after *T* → 0 in metallic systems.

In this work, only finite systems have been considered that have no partially occupied orbitals in the limit *T* → 0. For each system size, the conventional closed-shell zero-temperature ldrCCD calculation converges and agrees with the *T* → 0 limit of the finite-temperature ldrCCD calculation at the respective system size.^30^ If the finite-temperature SCF reference becomes degenerate for *T* → 0, as in system sizes deviating from the simple-cubic closed-shell numbers , finite-order thermal MBPT calculations diverge, as demonstrated by Hirata.^57^ ldrCCD calculations have been shown to converge to a finite value for such systems owing to the infinite-order resummation.^30^ Still, it remains to be studied whether an infinite-size extrapolation using degenerate system sizes, which deviate from the above closed-shell numbers, yields the same result as the thermodynamic limit extrapolations from closed-shell system sizes presented in this work. At zero temperature, it is a common practice only to choose closed-shell sizes. Extrapolations of finite-system calculations to the infinite-size limit agree well with continuous momentum integration-based methods that are in the thermodynamic limit by construction.^65,66^

At finite-temperature, only few resummed theories, such as the random phase approximation (RPA),^24^ can be integrated in momentum-space in practice, allowing the study of the *T* → 0 limit after the  limit in metallic systems. However, the only anomalous terms contained in direct-ring theories, such as RPA and ldrCCD, are particle/hole rings with zero momentum transfer ***q*** = 0. Including or excluding this point has no effect on the integral. Yet, there are more anomalous terms and whether their sum in other theories is zero, finite, or even diverging in the grand potential is an open question and topic of ongoing research. An alternative question is, how the number-conserving perturbation theory of Hirata and co-workers^55,56,67^ can be integrated in an infinite-order theory, such as RPA or coupled-cluster.

## 3 Numerical Results

To assess the large system-size estimates in the previous section, numerical calculations of the paramagnetic uniform electron gas have been conducted for simple-cubic systems containing 38, 54, 66, 114, 162, 246, 294, 342, 358, and 406 electrons on average. The system sizes have been chosen such that degenerate spatial orbitals can be fully occupied at zero-temperature in a closed-shell self-consistent field calculation.

### 3.1 Hartree Self-Consistent Field

Unlike in Hartree–Fock, where exchange is included in the SCF calculation, the metallic spectrum of the free uniform electron gas remains in the Hartree SCF. No band gap forms since the eigenenergies of mostly occupied and mostly unoccupied orbitals are affected in the same way. At zero temperature this has been found to be relevant for RPA-like calculations.^60^ Consequently, each eigenvalue in eq 6 only depends on its kinetic energy and the sum of all occupancies. A uniform shift of the eigenenergies Δε is the only number that needs to be found, although in a nonlinear equation. At finite temperature, all orbitals contribute in principle. In this work the number of spatial orbitals for the closed-shell SCF calculation has been truncated at roughly 800 times the number of orbitals occupied at zero temperature. Sums over the orbitals beyond this number occurring in Ω_0_ and *N*_H_ have been approximated by integrals. With this treatment, all SCF quantities are well converged and the computation time for the SCF calculation is still negligible compared to the correlation calculations. Note that the SCF calculations have been repeated to yield relaxed eigenenergies for each value of the chemical potential in search for the chemical potential μ_Hxc_ where the expected number of electrons matches the number of positive charges .

**Figure 2:**
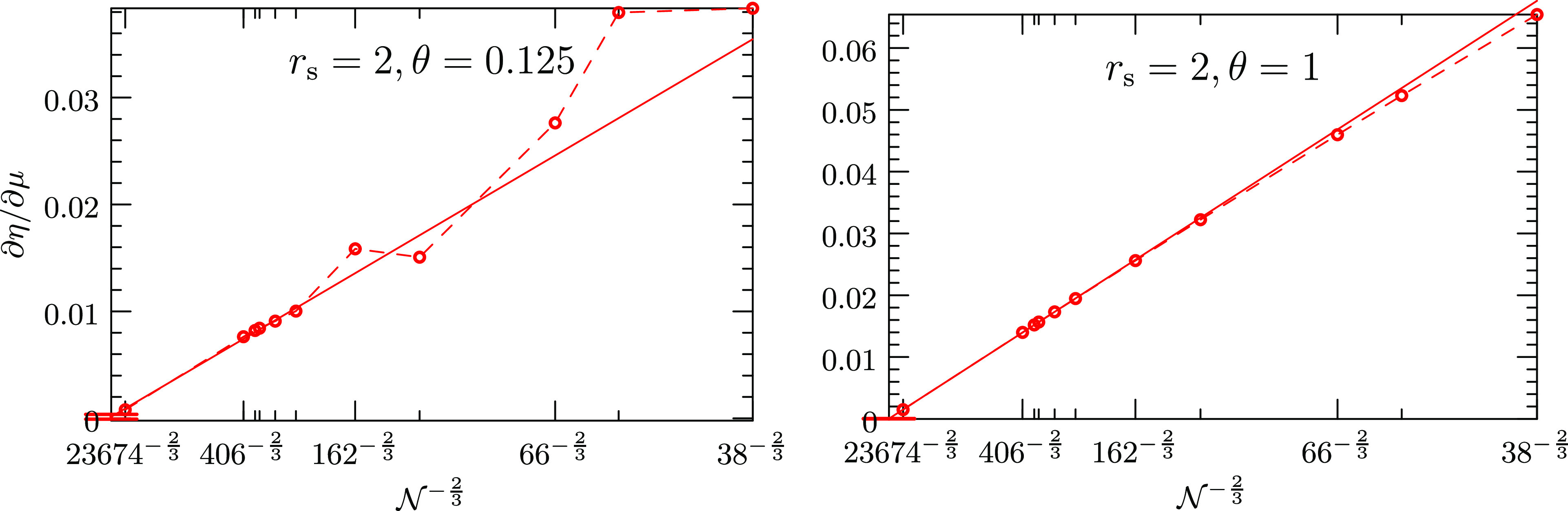
The effective potential η = μ – Δε is a measure for how quantities in the perturbation expansion depend on μ. The figure shows that the change of the effective potential η with respect to the chemical potential μ decreases with increasing system size. An extrapolation of the largest calculations with  indicates that the effective potential η becomes independent of μ in the thermodynamic limit.

Only the difference between the eigenenergies ε_*i*_ = ***k***_*i*_^2^/2 + Δε and the chemical potential μ occurs in the expressions of many-body perturbation theory where Δε depends on μ. Thus, they can be viewed rather as functions of the effective chemical potential η = μ – Δε. Figure 2 shows how the effective potential changes when the chemical potential is changed. It plots the derivative ∂η/∂μ against  where  is the system size. The derivative has been evaluated at the fully interacting chemical potential μ_Hxc_, except for the largest system size , where no correlation calculation has been conducted and μ_H_ has been used instead. Already for moderate system sizes, a change of the chemical potential has about 2 orders of magnitude less an effect on η and in consequence on the expressions of FT-MBPT. For large  the effect on η decreases, scaling as , as estimated in eq 29, and vanishes in the thermodynamic limit.

### 3.2 First-Order Exchange

The exchange contributions to the grand potential Ω_x_ have been evaluated according to eq 19 using all orbitals that have been considered in the SCF calculation. The convergence with the number of closed-shell orbitals is faster than for the SCF quantities and no analytic treatment of the orbitals beyond 800 times the zero-temperature orbitals is necessary. Although considerably more demanding computationally than the SCF calculation, its evaluation is still negligible compared to the correlation calculation. The derivative of the exchange contribution with respect to η for the expected number of electrons has been evaluated analytically.

### 3.3 Linearized Direct-Ring Coupled Cluster

The correlation and exchange effects beyond first order have been approximated on the level of closed-shell linearized direct-ring coupled cluster doubles (ldrCCD) theory. It is one of the simplest theories whose zero-temperature and infinite-size limit exists. Still, it is expected to capture the dominant part of the long-range correlation. The advantage of ldrCCD is that it can be evaluated from a diagonalization of an effective particle/hole Hamiltonian and consequently permits an analytic imaginary time integration. Apart from numerical considerations, the temperature can be arbitrarily low.

Let *N*_*p*_ denote the number of spatial orbitals considered for the ldrCCD calculation. The direct-ring structure of the effective Hamiltonian in eq 22 is momentum conserving. In a uniform system and due to point-group symmetry it is therefore sufficient to consider independent *N*_*p*_ × *N*_*p*_ matrices for each momentum difference vector ***q*** = ***k***_*b*_ – ***k***_*j*_ in the wedge 0 ≤ *q*_*x*_ ≤ *q*_*y*_ ≤ *q*_*z*_ instead of one *N*_*p*_^2^ × *N*_*p*_^2^ matrix. For the largest system size 114 independent 4385 × 4385 matrices have been diagonalized. The matrices are real valued and symmetric and can be diagonalized efficiently with standard linear algebra packages.

Unlike at zero-temperature, the spectrum of each matrix is not necessarily positive-definite. Negative eigenvalues Λ_*F*_^*F*^ can occur when eigenenergies of contributing hole-orbitals are above the energies of contributing particle-orbitals, which is possible at finite temperature. Negative eigenvalues pose numerical difficulties occurring in the exponent of eq 25. However, the final product with the square roots of the occupancy products  in eqs 23 and 24 leads to a finite contribution. In practice, the term δ_*a*_^*b*^δ_*j*_^*i*^Δ_*j*_^*b*^ has been truncated to zero if the occupancy and vacancy product *n*_*j*_^*b*^ was below 10^–12^.

For the ldrCCD calculation *N*_*p*_ has been chosen about 20 times the number of zero-temperature occupied spatial orbitals. The contribution from the orbitals beyond has been extrapolated from the asymptotic behavior of RPA-like correlation energies. The finite-basis-set error scales as , where *q*_max_ is the magnitude of the largest considered plane wave momentum difference.^68^ A Hann window has been used to obtain a soft cutoff for four different values of *q*_max_ to smooth the samples for the *q*_max_^–3^ extrapolation to the complete basis set (CBS) limit.^60^ The correlation coefficients of the regression curves range between 0.97 and practically 1. The 67% confidence intervals of the CBS limits are given in the ±CBS column in Table 1.

**Table 1:** Linearized Direct-Ring Coupled Cluster Doubles (ldrCCD) Exchange-Correlation Free Energies of the Spin-unpolarized (Paramagnetic) Warm Uniform Electron Gas for Various Densities and Temperatures^a^

*r*_s_	θ	*f*_xc_*r*_s_	Ω_x_*r*_s_/	Ω_c_*r*_s_/	±TDL	±CBS	*f*_xc_^iSTLS^*r*_s_	Δ*f*_xc_*r*_s_
2	0.125	–0.5421	–0.4278	–0.1143	±0.0020	±0.0010	–0.5444	+0.0023
	0.5	–0.4528	–0.2789	–0.1739	±0.0015	±0.0014	–0.5150	+0.0628
	1.0	–0.3852	–0.1739	–0.2113	±0.0025	±0.0014	–0.4650	+0.0789
8	0.125	–0.6186	–0.4279	–0.1907	±0.0050	±0.0041	–0.6304	+0.0118
	0.5	–0.5163	–0.2789	–0.2374	±0.0040	±0.0045	–0.6265	+0.1102
	1.0	–0.4522	–0.1739	–0.2752	±0.0050	±0.0046	–0.5992	+0.1470

a All energies are given in Hartree. *f*_xc_ is retrieved from the thermodynamic limit and complete-basis-set limit of Ω_xc_. Exchange and correlation contributions have been extrapolated separately. The expected statistical errors from the infinite-size and infinite-basis-set extrapolations of the correlation contributions are given in the ±TDL and ±CBS column, respectively. The TDL and CBS errors of the exchange contributions are negligible. For comparison, the free energies obtained from the improved Singwi–Tosi–Land–Sjölander^70^ parametrization of Quantum Monte Carlo results and the difference to ldrCCD are given in the columns *f*_xc_^iSTLS^ and Δ*f*_xc_, respectively.

For each system size, multiple calculations of Ω_Hxc_ have been conducted in search for the chemical potential μ_Hxc_ where *N*_Hxc_ = −∂_μ_Ω_Hxc_ agrees with the number of positive charges . The derivative of Ω_c_ has been evaluated numerically from a polynomial fit. The next estimate  at the current chemical potential μ has been found from the difference of  assuming that the dominant change in *N*_Hxc_ stems from the change in *N*_H_ = −∂_μ_Ω_0_. This gives an equation for the dominant change in the chemical potential
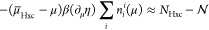
35where all involved quantities can be readily evaluated at the current chemical potential μ. This procedure has required about 8 iterations until convergence for each considered system size 

**Figure 3:**
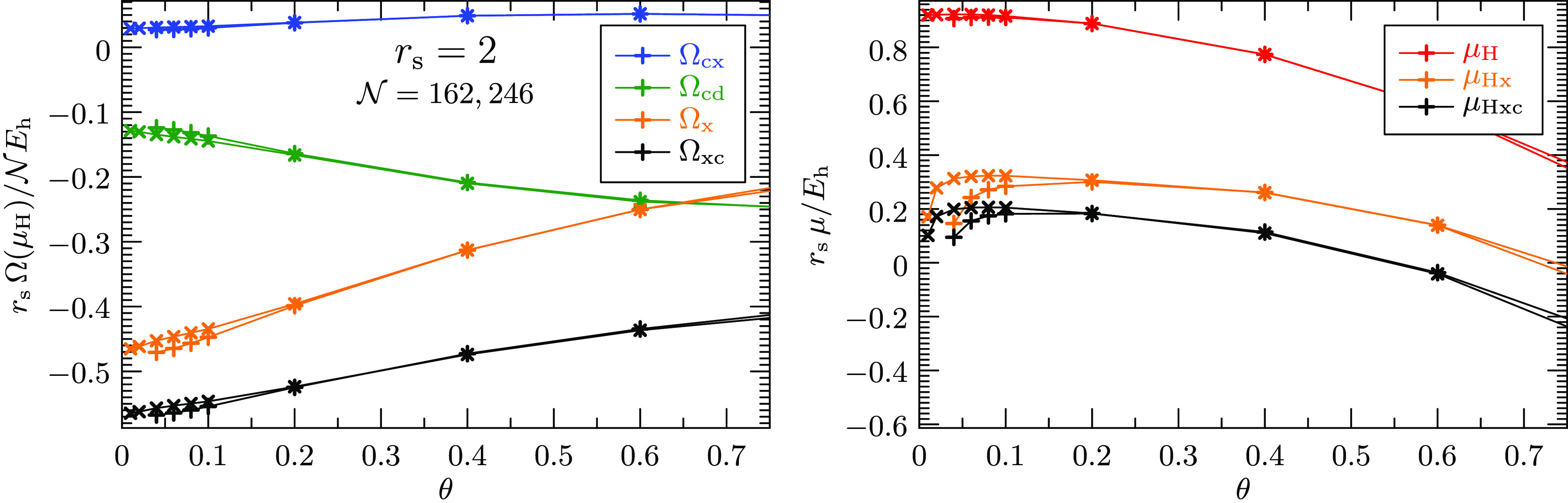
Left panel plots the contributions to the exchange-correlation grand potential Ω_xc_ = Ω_x_ + Ω_cd_ + Ω_cx_ as a function of the reduced temperature θ for two system sizes  and 246. Ω_x_ is the first-order exchange energy of eq 17, while Ω_cd_ and Ω_cx_ refer to the direct and exchange term of Ω_c_, stemming from the first and second diagram in eq 21, respectively. The right panel shows the chemical potentials satisfying the charge-neutrality condition at various levels of theory as a function of θ for the same system sizes. The  and  results are denoted by pluses (+) and crosses (×), respectively.

The left panel of Figure 3 depicts how the various contributions to the exchange-correlation grand potential Ω_xc_ depend on the reduced temperature for a fixed density of *r*_s_ = 2 and two system sizes  and . The right panel shows the temperature dependence of the chemical potentials satisfying the charge-neutrality condition at increasingly accurate levels of theory. The  and  results are denoted by pluses (+) and crosses (×), respectively. Deviations between these system sizes at low temperatures originate from a partition of the orbitals into (almost) purely occupied or purely unoccupied orbitals, occurring at lower temperatures for larger systems. A *T* → 0 extrapolation has not been conducted.

### 3.4 Free Energies in the Thermodynamic Limit

**Figure 4:**
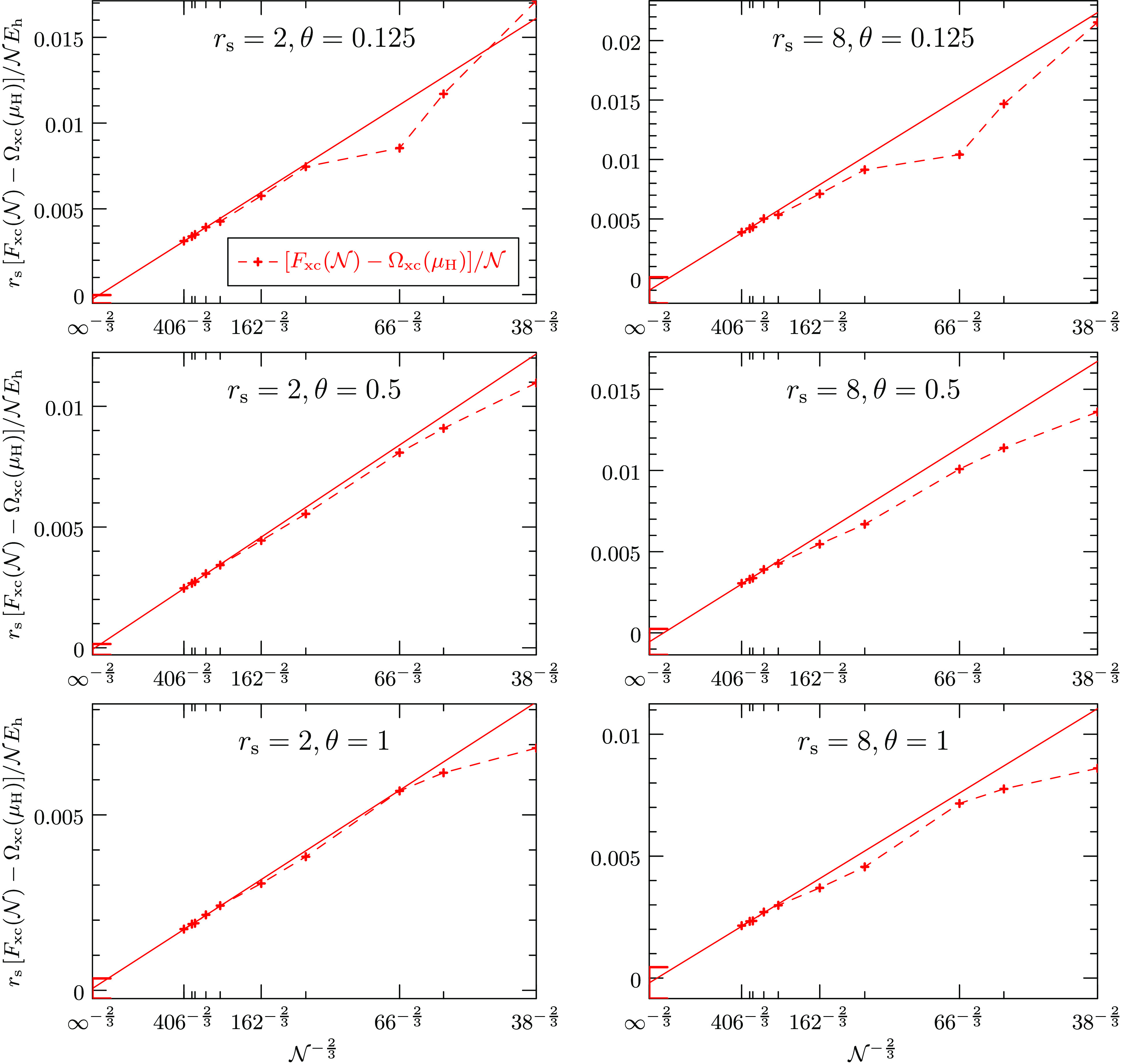
Finite-size dependence of the difference between the exchange-correlation free energy per electron and the exchange-correlation grand potential per electron for various densities and temperatures. The correlation contributions are approximated by the linearized direct-ring coupled cluster doubles (ldrCCD) theory. The grand potential has been evaluated at the noninteracting chemical potential μ_H_ while the free energy requires the correlated chemical potential μ_Hxc_. Extrapolations of the largest system sizes show that the two free energies coincide in the infinite-size limit.

Finding the thermodynamic limit poses a difficult task in the calculation of extended systems. First, we assess whether the asymptotic behavior estimated by eq 34 applies in the UEG as a prototypical warm-dense system. Figure 4 plots the difference between the exchange-correlation free energy per electron and the exchange-correlation grand potential per electron, evaluated at the Hartree chemical potential μ_H_, against the system size . In this graph, an asymptotic behavior as estimated from eq 34 is expected to appear as a line through the origin. As a guide to the eye, the results are connected with dashed red lines. The linear extrapolations from the largest system sizes are shown as solid red lines. The 67%-confidence intervals of the thermodynamic limits are indicated by the error bars on the vertical axis. They confirm numerically that the two exchange- correlation free energies agree in the thermodynamic limit of the warm UEG for all densities and temperature considered.

**Figure 5:**
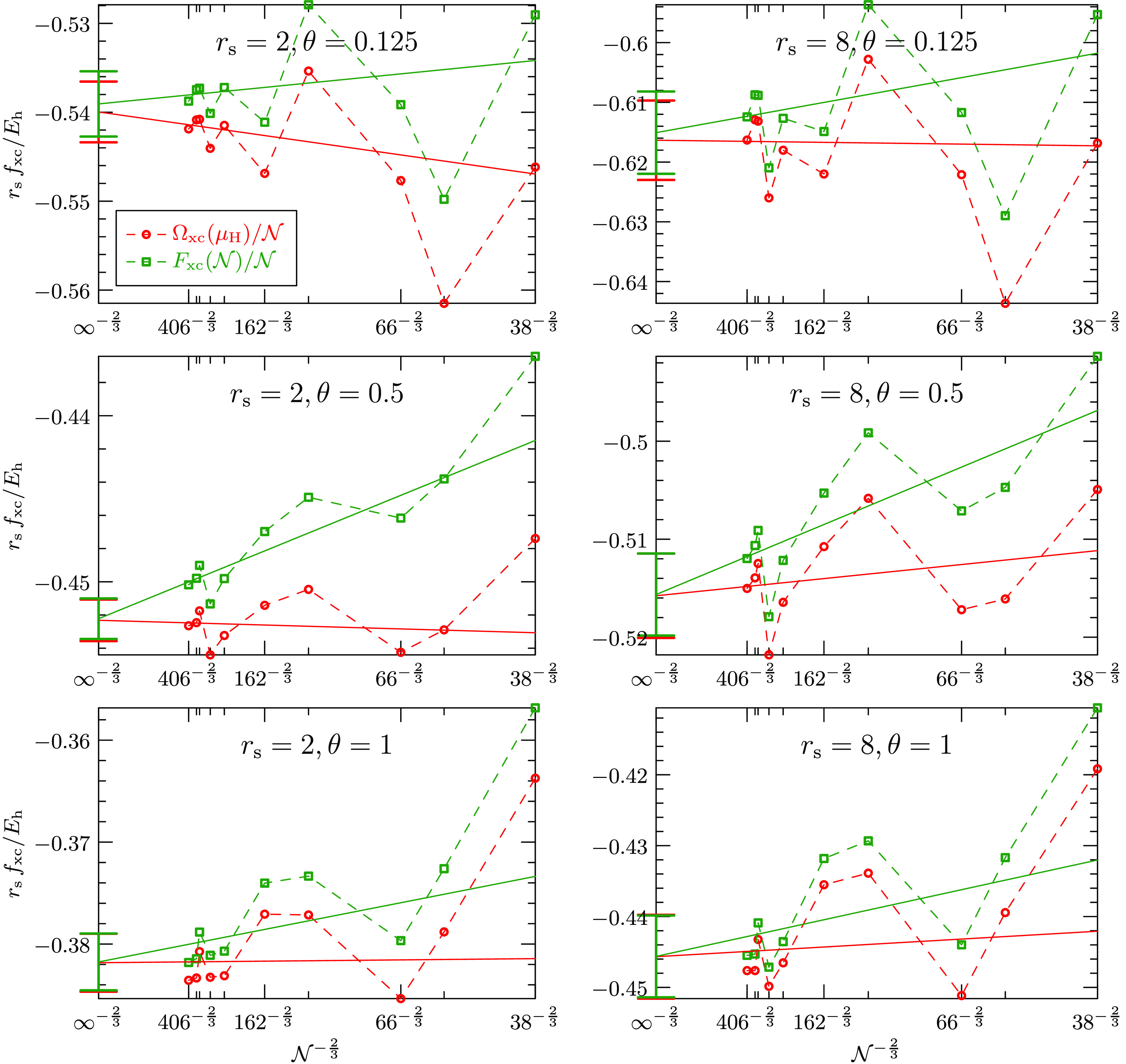
Finite-size dependence of the exchange-correlation grand potential Ω_xc_ per electron in red and the exchange-correlation free energy *F*_xc_ per electron in green for various densities and temperatures of the nonspin-polarized (paramagnetic) warm dense electron gas. The difference of the two graphs does not exhibit shell effects and is shown in Figure 4. The finite-size error of the exchange contribution is negligible for the largest system sizes and the remaining terms are expected to converge as  to the thermodynamic limit. The solid red and green lines show the extrapolations to the thermodynamic limit of the grand potential and the free energy, respectively. The error bars on the vertical axes indicate the statistical error of the extrapolations.

The terms in Ω_xc_ converge with different rates to the thermodynamic limit. At the largest considered system sizes the exchange contributions are almost converged. The remaining correlation terms converge as  in the low temperature regime and as  otherwise.^51^ Also, the effective chemical potential η, which Ω_c_ depends on, converges as . Thus, *F*_xc_ and Ω_xc_ are also individually expected to converge to the thermodynamic as . Figure 5 plots *F*_xc_ and Ω_xc_ individually against the system size , while Figure 4 showed only their difference. Unlike their difference, the two energies suffer from shell effects. They could be alleviated by twist averaging^51,69^ but this has not been done in this work. The solid lines show the  fit for the largest system sizes of the respective sets and the statistical error of the infinite-size extrapolation for both energies is indicated by the error bars on the vertical axes. Interestingly, in most cases the slope of the grand potential extrapolation is flatter than that of the free energy extrapolation, making the extrapolation of the grand potential less dependent on the functional form of the asymptotic behavior.

Table 1 summarizes the exchange-correlation free energies *f*_xc_ found in the thermodynamic limit and gives the 67% confidence interval of the infinite-size extrapolation in the ± TDL column. At low temperatures, the results compare well to the improved Singwi–Tosi–Land–Sjölander parametrization^70^ of previous Quantum Monte Carlo calculations despite the simple ldrCCD theory employed. For zero-temperature this fortuitous agreement of ldrCCD and also of drCCD has already been shown by Freeman.^65^ Reference (51) gives an overview over other parametrizations. At higher temperatures, however, the difference is sizable and ldrCCD underestimates the magnitude of the negative exchange-correlation free energy considerably.

**Figure 6:**
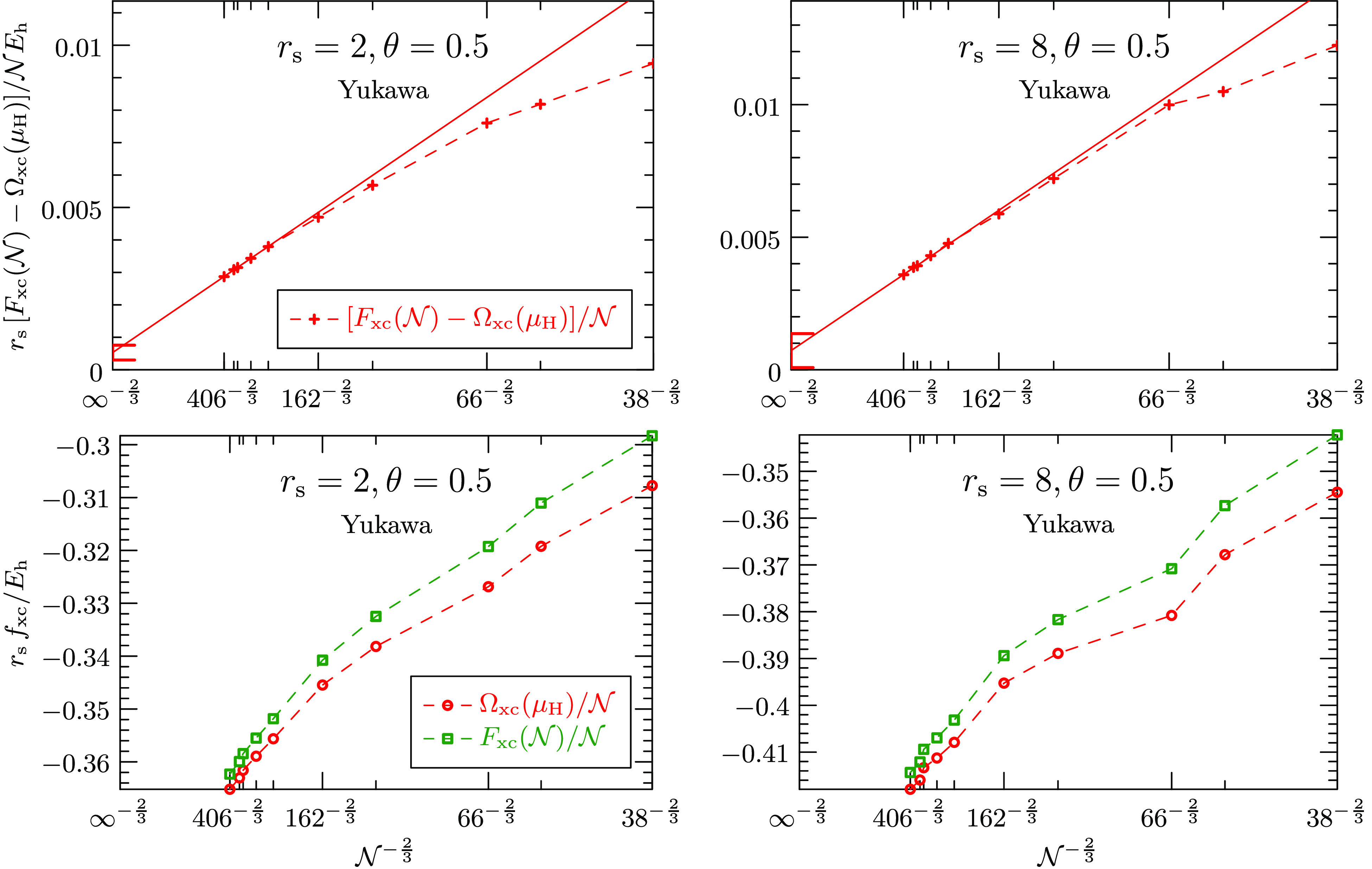
Top panels show the finite-size dependence of the difference between the exchange-correlation free energy and the exchange-correlation grand potential for θ = 0.5 and two densities employing the Yukawa regularization, rather than the Spencer–Alavi truncation. The bottom panels show these free energies individually. Although neither the exchange-correlation free energy nor the exchange-correlation grand potential have reached the asymptotic domain individually in case of the Yukawa regularization, their difference has—just barely: Only the largest 3 system sizes have been used for the extrapolation.

All of the previous numerical results have been produced employing the Spencer–Alavi spherical truncation scheme for all electrostatic interactions: the background–background interaction, the electron–background and the electron–electron interaction in the Hartree SCF, as well as the electron–electron interaction in the perturbation. To assess to what degree the estimates of the asymptotic behavior depend on the employed regularization scheme, the Yukawa regularization has been implemented as well and calculations have been conducted for various densities and temperatures. It can bee seen in the lower two panels of Figure 6 that the exchange-correlation free energy and the exchange-correlation grand potential individually hardly converge to the asymptotic domain using the Yukawa regularization. Note the different energy scales. Although only small shell effects are present, as opposed to the spherical truncation scheme, the Yukawa regularization is not useful in practice for retrieving converged results in the thermodynamic limit. In contrast, the difference of the two energies already exhibits an asymptotic behavior that agrees with the estimated behavior.

## 4 Summary

This work shows that the infinite-size limit of finite temperature many-body perturbation theory can be found efficiently with a truncated Coulomb interaction. The truncation radius is chosen such that the volume of the interaction agrees with the volume of the simulated cell. Such schemes have previously been studied in classical systems of electrostatically interacting particles, as well as for the Fock-exchange contribution in zero-temperature MBPT. Here, the truncation scheme is employed for all electrostatic interactions in the uniform electron gas: electron–electron, electron–background, and background–background.

It is found that due to the long-ranged nature of the electrostatic interaction the difference of the average number of mobile electrons and fixed positive charges scales asymptotically as  for large system sizes where  is the number of positive charges and μ_Hxc_ is the chemical potential where the number of electrons equals the number of positive charges, including exchange and correlation effects. Thus, the ratio of the number of electrons and positive charges tends to one for any finite choice of the chemical potential μ.

An important consequence is that also the exchange-correlation grand potential per electron, evaluated at the noninteracting Hartree self-consistent field chemical potential μ_H_, asymptotically agrees with the free energy per electron, found from a Legendre transformation at the interacting chemical potential μ_Hxc_:
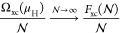
36The latter requires multiple iterations of the expensive correlation calculations during the nonlinear search for the interacting chemical potential for each system size considered for the thermodynamic limit extrapolation.

The above asymptotic behavior has been estimated in general for matter under warm-dense conditions and it has been shown explicitly for the warm uniform electron gas for various densities and temperatures employing the linearized direct-ring coupled cluster doubles theory for approximating exchange-correlation effects. The considered densities and temperatures cover the region where FT-MBPT methods, such as finite temperature coupled cluster, can complement other methods, such as quantum Monte Carlo methods.^52^

## Notes

The program and the settings used to produce the data in this work are publicly available at https://gitlab.cc4s.org/cqt/weg-ldrccd.git
